# An Experimental Inquiry Concerning the Reproduction of Nerves

**Published:** 1797

**Authors:** John Haighton


### XV. *An experimental Inquiry concerning the Reproduction of Nerves.* *By* John Haighton, *M. D.* Vide *Philosophical Transactions of the Royal Society of London, for the Year* 1795. Part I. 4to. London, 1795.

IN the preceding paper the reader has seen an account of the experiments made by Mr. Cruikshank, in the year 1776, and which induced him to believe that a nerve, when divided, possesses, like the bones and some other parts of an animal body, the power of repairing its loss with a substance resembling itself. Since that period different opinions* have prevailed among anatomists and physiologists concerning the nature of the new-formed substance; some maintaining that it is similar to the original nerve, while others suppose it to be totally different.

* *Vide* Fontana *Traité sur le Venin de la Vipere .... sur la Reproduction des Nerfs,* &c. Tom. II. 4to. Florence, 1781; and Arnemann *Versuche ueber das Gehirn und Ruckenmark.* 8vo. Goettingen, 1787.

When

When opinions ſo oppoſite to each other prevail on a point, which experiment ſeems ſo fully adequate to decide, we are naturally led, obſerves the ingenious author of the paper now before us, to take a view of the manner in which the experiments were conducted, and conſider the criterion to which each party has appealed.

There are only two teſts, Dr. Haighton remarks, which ſeem to offer themſelves, and from which any degree of judgment can be formed. Theſe are, either a minute and careful examination of the new-formed ſubſtance in an anatomical way, and an accurate comparifon of it with the original nerve; or, a cautious attention to the function of that nerve, by which we ſee the loſs of it from the diviſion, and the return of it from the reunion of the divided parts.

Thoſe who have ſubjected this matter to the teſt of experiment, have made their appeal to the firſt criterion; and have either affirmed or denied the reproduction, according as they thought the new-formed part either agreed with or differed from the original nerve.

This criterion, continues our author, certainiy ſuppoſes, that anatomy is fully competent to to determine what is the precise ſtructure of nerves, what are the nature and characters of ultimate nervous fibres, and by what mechaniſm or power they execute their allotted function. It ſuppoſes likewiſe, that anatomiſts are perfectly agreed upon this matter; and that thoſe who make their appeal to anatomy, have admitted a common ſtandard of compariſon, by which they allow their experiments to be judged; but no poſition, Dr. Haighton contends, can be more remote from fact than this is; for while ſome think ultimate nervous fibres are conſtructed to act by tremors, others believe them to be hollow tubes. Nor is the difference of opinion, he obſerves, leſs, reſpecting the appearances which they exhibit on being viewed by a microſcope: one eminent phyſiologiſt (Dr. Monro) ſuppoſing the ultimate nervous fibres to be "ſerpentine and con-"voluted;" while another ingenious inquirer, (Abbé Fontana) thought at one time that the fibres were compoſed of cylinders, with bands twined around them, in a ſpiral direction, till ſubſequent examination convinced him, that this appearance had its origin in an optical deception, and that their true direction was that of "parallel winding fibres."

As

As it appears then, that microſcopical obſervers neither agree with each other on this ſubject, nor with themſelves, our author thinks it fair to conclude, that ocular inſpection cannot be admitted as a fair appeal, from which we can determine whether the ſubſtance which unites the extremities of divided nerves is of the ſame nature as the original nerve. He therefore reſolved, he tells us, to ſubmit his inquiries to a teſt leſs doubtful and fallacious; and as ſuch a teſt was not to be found within the pale of anatomy, he was induced to try whether the reſources of phyſiology could not furniſh him with what he wiſhed.

From phyſiology, he obſerves, we learn, that *if the action of a nerve be ſuſpended by a diviſion of it, and if that action be recovered in conſequence of an union of its divided extremities, ſuch medium of union muſt poſſeſs the characters and properties of nerve.* He had therefore only to determine, what nerves appeared the moſt favourable for the experiment, and purſue the poſition juſt ſtated to its ultimate conſequence. "I know not," ſays he, "whether my choice "was judicious, but I determined on the eighth "pair."

The firſt ſtep he took in this inquiry, was to to aſcertain *what effects will ariſe from the diviſion of both of theſe nerves, together with that branch of the great ſympathetic nerve accompanying and ſtrongly adhering to them.*

### Experiment I.

A dog being properly ſecured, and a convenient inciſion made on the fore part of the neck, our author divided both the nerves of the eighth pair; the animal became immediately reſtleſs and uneaſy, betraying ſymptoms of great diſtreſs about the ſtomach, which continued eight hours, when he died.

Though the reſult of this experiment is perfectly agreeable to what other experimental phyſiologiſts have ſtated, Dr. Haighton thought it of importance, he tells us, to the preſent inquiry, to give it confirmation by farther experiment. He therefore repeated it on two other dogs, one of which ſurvived it three days, the other only two.

From theſe experiments, he obſerves, we learn, that the action of theſe nerves was ſuſpended, and that thoſe vital organs which received ceived their nervous energy from this fource, had their functions arrefted, fo that death followed as a neceffary confequence.

He is aware, it may be faid here, by way of objection, that a violent fhock had been fuddenly given to the machine; and that the animal perifhed rather from the fudden deprivation of the nervous influence, than from its abfolute lofs; and that if the fame quantity had been abftracted in a more gradual way, the animal might have furvived it. How little validity there would be in fuch an objection the following experiment, he thinks, will evince.

### Experiment II.

Another dog being procured, Dr. Haighton divided only one of the nerves of the eighth pair. He was furprifed to fee how flightly he was affected from it; for, excepting a little morofenefs, there was fcarcely any alteration, we are told, perceptible, fo that in a few hours after the operation he took food as ufual. On the the third day, our author divided the other nerve; but the ſame ſymptoms immediately ſupervened here as followed the diviſion of both nerves in the former experiments: the animal continued in a ſtate of reſtleſſneſs and anxiety, with palpitation and tremors, until the fourth day, when he died.

The event of this experiment, the author obſerves, differs in nothing more from the former, than that the fate of the animal was ſuſpended a little longer, but the ultimate effect was exactly the ſame: hence, he contends, that, in the firſt experiments, *the death of the animal is not to be imputed to the mere ſudden deprivation of nervous energy, but to its abſolute loſs.*

Wiſhing next to determine whether, by lengthening the interval between the diviſion of the two nerves, a few days more, the life of the animal could not be protracted to a greater length, or even ſaved, Dr. Haighton made the following experiment:

 Expe-

### EXPERIMENT III.

Having divided one of the nerves of the eighth pair, and waited the lapfe of nine days, he divided the other. The fame fymptoms, he obferves, came on now as in the laft experiment, but fcarcely fo violent. The only kind of food the animal would take was milk, and that in fmall quantities; and this, it feems, always produced great uneafinefs at the ftomach, with fymptoms of indigeftion. In this ftate he continued thirteen days, and then died, much emaciated.

From this dog having lingered fo long, Dr. Haighton tells us he was beginning to entertain hopes of his recovery, and had that eventually happened, he doubted much whether, even under the prefent uncertainty of things, he could have refifted the temptation of afcribing fuch recovery to the reproduction of the nerves; but the event, he adds, put a ftop to his fpeculation.

Having thus, as he thinks, proved his firft pofition, that whether the eighth pair of nerves be be divided in immediate fucceffion, fo as to deprive an animal of their influence fuddenly, or whether this deprivation be effected in a more gradual way, the confequences are in the end equally fatal; Dr. Haighton next endeavours to avail himfelf of this fact in the folution of the problem now before us. If, he obferves, the fubftance of nerve be reproduced, certainly a period longer than the above muft be neceffary for the procefs; but to mark the precife point of time when the line is to be drawn, would, he adds, require the facrifice of more animals than a queftion of mere curiofity could juftify. He therefore contents himfelf with giving a general anfwer to the queftion, and inquiring whether, by fufpending the divifion of the fecond nerve for a much greater length of time than was done in the two laft experiments, the exiftence of the animal could be preferved.

### Experiment IV.

Having procured another dog, and divided one of the nerves of the eighth pair, Dr.

Haighton allowed fix weeks to elapfe before the other was cut through. This divifion of the correfponding nerve, it is obferved, evidently deranged the animal; but in a much lefs degree than in the former experiments. For fome days, we are told, he refufed folid food, but took milk; afterwards he ate folid food in fmall quantities, and near a month had paffed away before he fed as ufual. The actions of the ftomach, it is added, were for a long time evidently deranged, fo that he was continually haraffed with fymptoms of indigeftion; and fix months had nearly elapfed before he recovered his health, though during five months of the time he took his ufual quantity of food.

Now, to what caufe, our author afks, are we to impute the recovery of the animal in this experiment? The moft probable one he thinks is, that in the interval of fix weeks the firft nerve had been reproduced; fo that the actions of the organs depending upon this nerve, though fomewhat difturbed, were not fufpended: and afterwards, as the union of the fecond nerve advanced, and the reproduction of the firft became more perfect, the vital organs gradually recovered their healthy ftate.

Dr. Haighton kept this animal nineteen months, months, during the greateſt part of which time he performed the office of a yard dog. And here he has thought it proper to obſerve, that in all the experiments, the voice was totally loſt on the diviſion of the ſecond nerve. This effect, anatomiſts, he adds, will eaſily underſtand, from recollecting that the recurrent branches of the eighth pair, which are the true vocal nerves, originate below the part where the trunks of the eighth pair were cut through; conſequently thoſe nerves were themſelves in effect divided. Now it deſerves to be remarked, continues our author, that the dog's voice returned in proportion as his general health improved; and that in about ſix months he could bark as ſtrongly as before, but that the pitch of his voice was evidently raiſed.

From this experiment, Dr. Haighton is ſtrongly inclined to believe that there muſt have been a true reproduction of the nerve; yet he does not contend, that if the part of union were examined by an anatomical eye, ſuch reproduction would be very evident. On the contrary, he is perſuaded that anatomy can determine only the preſence and exiſtence of an uniting medium; but it is the province of phyſiology, he obſerves, to decide whether the medium of union poſſeſs the characters, and perform the function, of the original nerve.

He is aware that the evidence of reproduction, as reſting on this experiment, may not be ſufficient to obviate every doubt which reflections upon this ſubject may probably ſuggeſt; and that ſome may be diſpoſed to object to the poſſibility of the ſtomach and larynx having received an additional ſupply of nervous energy from other ſources; eſpecially as the former of theſe is known to receive nerves from the great ſympathetic as well as from the eighth pair; and the larynx receives a branch from this (the eighth) pair, which ariſes from the trunk above the part where the diviſion was made. Our author candidly acknowledges that the familiar analogy of the vaſcular ſyſtem, where collateral branches are enlarged from the obliteration of a principal trunk, tends farther to give weight to theſe doubts.

To remove theſe ſeeming difficulties by anatomical inveſtigation, or by directing his views to any changes that might be induced on the anaſtomoſing nervous filaments, would, he obſerves, be an undertaking not leſs tedious in its execution than unſatisfactory in its reſult; for there would ſtill remain room for oppoſite opinions; nions: and while fome would argue that thefe anaftomofing filaments were become evidently enlarged, others would contend that they had not fuffered the flighteft change.

Now, our author, as we have feen, has already expreffed his diftruft of thofe decifions which are founded on an appeal to the eye, feeing that anatomy has yet to explain by what mechanifm or ftructure thefe organs perform their office. He therefore has preferred an appeal to the functions of thefe parts, inquiring whether, in the experiment in which the dog furvived the divifion of the fecond nerve of the eighth pair after an interval of fix weeks, it was effected by the reproduction of the firft divided nerve, or in another way?

There are only two poffible anfwers, he obferves, to fuch a queftion; thefe are, that either the functions of the ftomach, larynx, &c. were carried on by anaftomofing nerves; or that the united nerves had recovered their original importance.

If the firft be contended for, this confequence, he thinks, ought to enfue, viz. that the eighth pair fhould now be entirely ufelefs, and both of them may be divided a fecond time, without injuring any of the functions of the animal.

If the laſt be granted, it muſt, he contends, of neceſſity follow, that the medium of union poſſeſſed the ſame properties as the original nerve.

He has thus, he thinks, circumſcribed the field of inquiry, and drawn the queſtion into ſo narrow a compaſs, that it is in the power of a ſingle experiment to prove either the affirmative or negative. If now the eighth pair be divided a ſecond time in immediate ſucceſſion, and the animal ſuſtain it with impunity, he conceives it right to conclude, that the actions of thoſe organs, which originally were carried on through the means of the eighth pair, are now performed by other channels, and that the true ſubſtance of the nerve is not reproduced. But, on the contrary, if the animal die in conſequence of it, then he thinks it equally juſt to infer, that the new-formed ſubſtance is really and truly *nerve*, becauſe we know of no other ſubſtance which can perform the office of nerve.

He relies, therefore, he tells us, upon the following, and conſiders it as his *experimentum crucis*.

### Experiment V.

Having the dog in his poffeffion upon which he divided the eighth pair of nerves nineteen months before, he cut through both of them now, in immediate fucceffion. The ufual fymptoms, we are told, were immediately induced, and continued until the fecond day, when he died.

After death Dr. Haighton carefully diffected out thefe nerves, and has preferved them as evidence of his fuccefs. He thinks he has now anfwered the queftion he has propofed to himfelf, and proved that nerves are not only capable of being united when divided, but that the new-formed fubftance is really and truly nerve.

