# Errata in Vol. VII

**Published:** 1800

**Authors:** 


					i55? .line 5?> for " 1793" read " 1795."

				

